# Tumor suppressors BTG1 and BTG2: Beyond growth control

**DOI:** 10.1002/jcp.27407

**Published:** 2018-10-23

**Authors:** Laurensia Yuniati, Blanca Scheijen, Laurens T. van der Meer, Frank N. van Leeuwen

**Affiliations:** ^1^ Laboratory of Pediatric Oncology, Radboud Institute for Molecular Life Science, Radboud University Medical Center Nijmegen The Netherlands; ^2^ Hubrecht Institute–KNAW, University Medical Center Utrecht Utrecht The Netherlands; ^3^ Department of Pathology Radboud Institute for Molecular Life Sciences, Radboud University Medical Center Nijmegen The Netherlands

**Keywords:** antiproliferation, B‐cell malignancies, BTG1, BTG2, tumor suppressor

## Abstract

Since the identification of B‐cell translocation gene 1 (BTG1) and BTG2 as antiproliferation genes more than two decades ago, their protein products have been implicated in a variety of cellular processes including cell division, DNA repair, transcriptional regulation and messenger RNA stability. In addition to affecting differentiation during development and in the adult, BTG proteins play an important role in maintaining homeostasis under conditions of cellular stress. Genomic profiling of B‐cell leukemia and lymphoma has put *BTG1* and *BTG2* in the spotlight, since both genes are frequently deleted or mutated in these malignancies, pointing towards a role as tumor suppressors. Moreover, in solid tumors, reduced expression of BTG1 or BTG2 is often correlated with malignant cell behavior and poor treatment outcome. Recent studies have uncovered novel roles for BTG1 and BTG2 in genotoxic and integrated stress responses, as well as during hematopoiesis. This review summarizes what is currently known about the roles of BTG1 and BTG2 in these and other cellular processes. In addition, we will highlight the molecular mechanisms and biological consequences of BTG1 and BTG2 deregulation during cancer progression and elaborate on the potential clinical implications of these findings.

## 1 INTRODUCTION

The B‐cell translocation gene (BTG)/TOB family of antiproliferation proteins regulates cell‐cycle progression, apoptosis, and differentiation. In particular, BTG1 and BTG2 have been identified as mediators of genotoxic and cellular stress signaling pathways, promoting either cell death or survival. Moreover, a role for BTG1 and BTG2 as tumor suppressors in both lymphoid malignancies and solid tumors is emerging. The capacity of BTG1 and BTG2 to protect cells from oncogenic transformation relates to their ability to regulate gene expression through association with transcriptional cofactors, but also at the posttranscriptional level by controlling messenger RNA (mRNA) stability. Furthermore, there is evidence that expression levels of BTG1 and BTG2 can be used as prognostic biomarkers in various cancers.

## 2 THE STRUCTURE AND TRANSCRIPTIONAL REGULATION OF BTG PROTEINS

**Figure 1:**
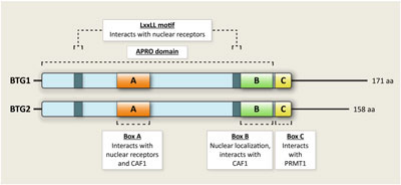
Domains and interaction partners of BTG1 and BTG2. The conserved core of BTG1 and BTG2, known as the APRO domain, contains three smaller motifs, known as box A, box B, and box C. These boxes facilitate interactions with various protein partners. An additional LxxLL motif, which is required for binding to nuclear receptors, is also found in BTG1 and BTG2. BTG: B‐cell translocation gene; CAF1, CCR4‐associated factor 1; PRMT1, protein arginine methyltransferase I [Color figure can be viewed at wileyonlinelibrary.com]

*BTG1* was first identified as a translocation partner of the c‐MYC gene in a case of B‐cell chronic lymphocytic leukemia (Rimokh et al., 1991). Soon thereafter it was found that *BTG1* expression varied during cell‐cycle progression and that overexpression of its gene product led to a cessation of growth, leading to the term “antiproliferation gene” (Rouault et al., 1992). The highly related *BTG2* gene was discovered around the same time as a gene rapidly induced by growth factors and mitogens (Bradbury, Possenti, Shooter, & Tirone, 1991; Fletcher et al., 1991). A third member of this family, TOB1 was identified a few years later, showing structural and functional similarities to BTG1. The subsequent inclusion of three other proteins sharing a conserved core known as AntiPROliferative (APRO) domain, as well as antiproliferative properties, makes up what is currently known as the BTG/TOB protein family. These six related proteins are known as BTG1, BTG2/PC3/Tis21, BTG3/ANA, BTG4/PC3B, TOB1/TOB, and TOB2 (Winkler, 2010). The availability of crystal structures for BTG2 and TOB1 confirms the presence of a structurally conserved region within this protein family, harboring two motifs known as box A and box B. A third motif, box C, is exclusively found in BTG1 and its closest relative BTG2. The latter two genes, which are the focus of this review, share 66% identity at the amino acid level; the only substantial difference is the slightly longer C‐terminal region of BTG1 (Rouault et al., 1992). Two LxxLL motifs, known to facilitate protein–protein interactions, are located in the core region of BTG1 and BTG2 (Figure 1).

The human *BTG1* and *BTG2* genes are located on chromosomes 12q22 and 1q32, respectively, and made up of only two exons (Rimokh et al., 1991; Rouault et al., 1992, 1996). The resulting transcripts and proteins encoded are highly unstable. Both BTG1 and BTG2 protein stability is regulated by the proteasome, which involves ubiquitination by the SCF–βTrCP1 complex (Sasajima, Nakagawa, Kashiwayanagi, & Yokosawa, 2012). BTG2 protein stability is also controlled by the SCF–Skp2 complex (T. J. Park, Kim, Park, Kim, & Lim, 2009). Moreover, BTG1 and BTG2 are subject to other posttranslational modifications. For instance, both proteins are phosphorylated at specific serine residues, allowing interactions with other cellular effectors. It was shown recently that mRNA expression of both *BTG1* and *BTG2* is subject to regulation by microRNAs. For instance, c‐MYC can suppress *BTG1* through *miR‐17–92* to maintain sustained proliferation and a neoplastic state in lymphoma cells (Li, Choi, Casey, Dill, & Felsher, 2014). As many as 17 binding sites for miRNAs are found in the 3′ untranslated regions of *BTG2*, suggesting a major role for these molecules in controlling BTG2 transcript levels (Fei, Haffner, & Huttner, 2014).

BTG1 and BTG2 are present both in the nucleus and the cytoplasm, and it has been suggested that the cellular trafficking of both proteins influences their activities (Kawakubo et al., 2006; Rodier et al., 2001). Overall, the COOH‐terminal region regulates nuclear localization, while the NH_2_‐terminal part is required for cytoplasmic retention. Although both genes are broadly expressed, the BTG1 expression is most abundant in the pancreas, heart, and hematopoietic tissues, while high levels of BTG2 are detected in various organs including kidney, lung, prostate, pancreas, thymus, central nervous system, and skeletal muscle. Consistent with their antiproliferative role, expression appears to be highest in quiescent cells, and downregulated when cells progress towards the G1–S transition (Rouault et al., 1992, 1996).

*BTG2* was originally identified as a p53‐inducible gene. Expression of *BTG2* is significantly increased in response to DNA damage, and this increase is a consequence of p53 induction since the expression of a loss‐of‐function p53 mutant does not lead to BTG2 accumulation in this context (Rouault et al., 1992). *BTG2* was also shown to be sensitive to nuclear factor*‐*κB (NF‐κB) activation. The BTG2 expression is induced by a variety of genotoxic agents (ionizing radiation, UV, adriamycin), growth factors, estrogen, serum, tetradecanoylphorbol acetate, interleukin 6, and cyclic adenosine monophosphate (cAMP). *BTG1* is also DNA damage‐inducible gene, but unlike *BTG2*, *BTG1* induction appears to be independent of p53 (Cortes et al., 2000). BTG1 transcript levels are elevated in response to glucocorticoid exposure, 4‐hydroxytamoxifen, triiodothyronine (T3), transforming growth factor β (TGF‐β), serum and angiogenic growth factors. Hence, BTG1 and BTG2 are subject to regulation by a variety of steroid hormone receptors and growth factor pathways.

## 3 BTG1 and BTG2 FUNCTION AS GLOBAL REGULATORS OF GENE EXPRESSION

### 3.1 Regulation of gene transcription

Both BTG1 and BTG2 function as transcriptional coactivators that associate with various cellular targets. For instance, both proteins can bind to and positively modulate the activity of HoxB9, a member of Hox gene family of transcription factors, critical determinants of pattern formation during metazoan development (Prevot et al., 2000). Moreover, the two conserved LxxLL motifs found in both BTG1 and BTG2 allow interaction with and modulation of various nuclear receptors, including T3 receptor, all‐trans retinoic acid (RA) receptor, estrogen receptor α (ERα) and androgen receptor (Busson et al., 2005; Hu et al., 2011; Prevot et al., 2001). The box C domain, exclusively present in BTG1 and BTG2 (Figure 1), facilitates binding to protein arginine methyltransferase I (PRMT1; Lin, Gary, Yang, Clarke, & Herschman, 1996). Members of this enzyme family catalyze arginine methylation on both histone and nonhistone proteins. This type of posttranslational modification, which is abundantly found in mammalian tissues, has been implicated in various biological processes (e.g., signaling events, transcription, mRNA biogenesis) and can become deregulated in cancer cells (Bedford & Clarke, 2009). PRMT1 is the primary enzyme mediating asymmetric dimethylarginine methylation. One of the most well‐studied biological roles for PRMT1‐mediated arginine methylation is to coactivate transcription. Consistent with these findings, BTG2, together with PRMT1, enhances RA transcription activity and RA‐induced differentiation (Passeri et al., 2006), while we have demonstrated that the BTG1–PRMT1 complex positively regulates glucocorticoid receptor (GR) signaling in leukemic cells (van Galen et al., 2010). Similarly, BTG1 was found to improve insulin sensitivity by promoting RAR activation and, eventually, c‐Jun‐mediated transcription (Xiao et al., 2015), which adds to the previously reported role of BTG1 as an enhancer of c‐Jun transcriptional activity during muscle development (Busson et al., 2005).

### 3.2 Posttranscriptional regulation

In addition to their involvement in transcriptional regulation, BTG family members affect gene expression by controlling mRNA abundance. Both BTG1 and BTG2 were shown to bind to the Ccr4‐associated factor 1 (CAF1) subunit of the multisubunit CCR4–NOT complex. This deadenylase complex promotes mRNA degradation by shortening/removal of the poly(A) tail (Mauxion, Faux, & Seraphin, 2008; Rouault et al., 1998). BTG2, through its interaction with CAF1 and CCR4, enhances mRNA deadenylation and consequently mRNA decay (Mauxion et al., 2008). More specifically, BTG2 binds to the poly(A)‐binding protein PABPC1 to stimulate CAF1 deadenylase activity, thus directly controlling poly(A) tail length (Stupfler, Birck, Seraphin, & Mauxion, 2016). The CNOT7 and CNOT8 deadenylase subunits of the CCR4–NOT complex are also bound by BTG proteins, affecting mRNA turnover of several genes, although the exact mechanism is unknown (Aslam, Mittal, Koch, Andrau, & Winkler, 2009). In addition, BTG2 expression, under the coordination of the microRNA miR‐132, represses the translation of specific circadian clock‐related proteins by enhancing their mRNA decay (Alvarez‐Saavedra et al., 2011).

The functional consequences of BTG1/2‐induced deadenylation in vivo remain incompletely understood. Since the poly(A) tail not only maintains mRNA stability but also regulates protein translation, changes in poly(A) tail length could also lead to a block in protein translation. It is presently unclear to what extent control of deadenylation contributes to the various cell biological processes affected by BTG1 or BTG2.

## 4 BIOLOGICAL PROCESSES REGULATED BY BTG PROTEINS

**Figure 2:**
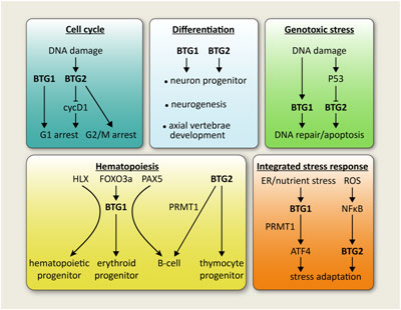
Major biological processes regulated by BTG1 and BTG2. Five major biological processes regulated by BTG1 and BTG2. Cell cycle**:** BTG1 and BTG2 expression induce cell cycle arrest at the G1 stage. BTG2 also facilitates DNA damage‐induced G2/M arrest. Differentiation: BTG1 and BTG2 expression are crucial for the differentiation of various tissues such as neurons and axial skeleton. Genotoxic stress: DNA damage can lead to programmed cell death via BTG1 and BTG2, in‐or dependent of p53. Integrated stress response: BTG1, together with PRMT1, promotes ATF4‐mediated cellular stress adaptation. BTG2 is a downstream effector of ROS and NF‐κB to overcome oxidative stress. Hematopoiesis: BTG1 acts as downstream effector of HLX, FOXO3a, and PAX5 to regulate the differentiation of hematopoietic, erythroid and B‐cells progenitors, respectively. BTG2 is involved in the differentiation of B cells and thymocyte progenitors. ATF4: activating transcription factor 4; BTG: B‐cell translocation gene; HLX: H2.0‐like homeobox; NF‐κB: nuclear factor‐κB; ROS: reactive oxygen species [Color figure can be viewed at wileyonlinelibrary.com]

Both BTG1 and BTG2 act as effectors of signaling pathways that take part in the regulation of key cellular processes, such as differentiation and apoptosis. BTG1 and BTG2 are both negative regulators of the cell cycle, and in some cell types, their overexpression can lead to cell death. Next to this, BTG1 and BTG2 expression are required for the differentiation of neuronal cells, the proliferation of myoblasts, development of vertebral patterning, and maintenance of hematopoietic progenitor cells. Cellular response to genotoxic stress requires BTG2, which acts in response to p53 activation. BTG1 is a novel component of the ISR, positively regulating activating transcription factor 4 (ATF4)‐mediated transcriptional activity in response to cellular stress conditions. The major biological processes affected by BTG1 and BTG2 function are summarized in Figure 2.

### 4.1 Regulation of cell cycle and apoptosis

The first evidence linking BTG1 to the control of cell growth and division came from the observation that its transcript levels peak in the G0/G1 phase of cell cycle and decrease dramatically during the G1/S phase transition (Rouault et al., 1992). Consistent with this notion, overexpression of BTG1 generally suppresses cell growth. However, how BTG1 expression contributes to the tightly controlled cell‐cycle transition remains to be elucidated. More is known about the role of BTG2 during cell‐cycle progression. Similar to *BTG1*, *BTG2* mRNA levels are highest in quiescent cells, and forced expression of this gene leads to suppression of growth (I. K. Lim et al., 1998; Montagnoli, Guardavaccaro, Starace, & Tirone, 1996). Synchronization experiments revealed that the antiproliferative effect of BTG2 involves downregulation of cyclin D1, leading to the inhibition of retinoblastoma (Rb) phosphorylation and G1 arrest (Guardavaccaro et al., 2000; Montagnoli et al., 1996). This suppression of cyclin D1 levels was recently shown to be dependent on the binding between BTG2 and histone deacetylases HDAC1, HDAC4, and HDAC9 (Micheli, D'Andrea, Leonardi, & Tirone, 2016). In the absence of functional Rb, BTG2 prevents G1 to the S phase progression by reducing the level of cyclin E and cyclin‐dependent kinase (cdk) 4 (I. K. Lim et al., 1998). More specifically, recent work showed that in B cells, cdk4 is a direct target of the BTG2–PRMT1 complex and that its methylation results in degradation of the protein (Dolezal et al., 2017).

Moreover, BTG2 is capable of inducing a G2/M arrest in a p53‐independent manner. BTG2 expression appears to be sufficient to induce cellular senescence in normal fibroblasts by antagonizing the cell‐cycle regulator Pin1 (Wheaton, Muir, Ma, & Benchimol, 2010). In human tumor cell lines, one feature of drug‐induced cellular senescence is upregulation of BTG1 and BTG2, with no or limited dependence on p53 expression (Chang et al., 2002). Finally, four members of the B‐cell translocation gene family (BTG1, BTG2, BTG3, and TOB1) were found to be regulated by the tumor suppressor p19(Arf), in a p53‐independent manner, leading to cell‐cycle arrest (Kuo et al., 2003).

Of note, BTG1 and BTG2 expression may not only lead to induction of cell‐cycle arrest but may also be involved in the control of apoptosis. Forced expression of BTG1 leads to increased cell death in several cell types including murine fibroblasts, microglia, and human breast cancer cells. In the brain, upregulation of BTG1 sensitizes microglial cells to inflammatory‐induced death (Lee et al., 2003). In breast tissue, apoptosis, induced by suppression of the antiapoptotic protein BCL2, requires expression of BTG1 (Nahta et al., 2006), while in atherosclerotic lesions, BTG1 expression localizes to macrophage‐rich areas as well as apoptotic cells (Corjay, Kearney, Munzer, Diamond, & Stoltenborg, 1998).

### 4.2 Cellular differentiation

Owing to their roles in controlling cell growth through regulation of cell‐cycle transition or arrest, both BTG1 and BTG2 exert unique functions during differentiation and maintenance of certain tissues. For instance, BTG1 expression appears to be required for the maintenance of stem and progenitor cells in the brain. In mice lacking Btg1 expression, the proliferating dentate gyrus stem and progenitor cells decreased significantly by number and underwent apoptosis. This phenomenon was observed in both young and adult *Btg1*‐null mice. Taken together, loss of BTG1 negatively affects the proliferation and induces apoptosis of these cells in the dentate gyrus and subventricular zone (Farioli‐Vecchioli, Micheli, et al., 2012). BTG2 plays a role in the neurogenesis during adulthood; its expression level is induced during neurogenesis and inhibition of expression leads to the programmed death of differentiated neurons in vitro (el‐Ghissassi et al., 2002). In vivo, mice deficient for *Btg2* show an accumulation of undifferentiated neurons and impaired contextual memory. This may be the consequence of *Btg2* being the negative regulator of *Id3*, an inhibitor of proneural gene activity (Farioli‐Vecchioli et al., 2009). How BTG proteins impinge on neuronal developments remains poorly understood. One study suggests that BTG2, together with the arginine methyltransferase PRMT1, controls neurite outgrowth by regulating arginine methylation in the nucleus (Miyata, Mori, & Tohyama, 2008). In differentiated neuronal cells, BTG2 is critical for neuroprotection as an effector of the transcription factor cAMP‐response element binding protein (CREB; Tan, Zhang, Hoffmann, & Bading, 2012). In addition, BTG1 was shown to be involved in differentiation and proliferation of myoblasts, endothelial cells, sperm cells and ovary cells. BTG2 also participates in myoblast proliferation and differentiation by regulating cyclin D1 levels (Evangelisti et al., 2009). BTG2 expression, under control of Stat3 signaling, also regulates adipocyte differentiation (S. Kim, Hong, & Park, 2016).

In vivo, Btg2 transcript levels are regulated during pregnancy, lactation and involution in the rat mammary gland (Kawakubo et al., 2004), consistent with a role in proliferative control during mammary gland development. Moreover, studies using *Btg2* knockout mice revealed that Btg2 expression is indispensable for the development of the axial vertebrae since these mice exhibited abnormal vertebral patterns. As *Btg2* was also found to be a positive regulator of the bone morphogenetic protein (BMP) signaling pathway, this vertebral transformation in *Btg2*‐null mice was proposed to be a consequence of attenuated BMP signaling (S. Park et al., 2004). We recently demonstrated, using *Btg1* knockout mice, that Btg1 expression also contributes to the normal vertebral patterning of the axial skeleton. Deletion of *Btg1* gene resulted in the partial posterior transformation of the seventh cervical vertebra, and this defect is enhanced by losing both *Btg1* and *Btg2*. *Btg2*‐deficient mice also showed impaired development in the thoracic–lumbar region of the axial skeleton and exhibited posterior homeotic transformation at the thoracic–lumbar junction, which was not observed in *Btg1*‐deficient mice. In conclusion, while loss of Btg2 has more pronounced effects on posterior transformation, Btg1 fulfills both unique and synergistic roles in maintaining the identity of the axial skeleton (Tijchon et al., 2015).

### 4.3 Hematopoiesis

The development and maintenance of hematopoietic stem cells (HSCs) is tightly controlled by a hierarchy of transcription factors, which are subject to regulation by complex signaling cascades. HSCs are defined by their predominantly quiescent state, their capacity to generate lineage‐committed progenitors, and their ability to be “active” and self‐renew in response to stress insults, such as chemotherapy intervention. During stress‐induced activation of HSCs, the BTG1 expression is required to return from a proliferative state back into quiescence (Venezia et al., 2004). Furthermore, *BTG1* is among the downstream effectors of nonclustered H2.0‐like homeobox (HLX), which acts as an important regulator of early hematopoiesis (Kawahara et al., 2012). Next, to its role in HSCs, BTG1 expression was found to regulate the expansion and differentiation of erythroid progenitor cells downstream of the transcription factor FOXO3a (Bakker et al., 2004).

BTG2 is also involved in both proliferation and differentiation of hematopoietic cells. During the course of thymocyte development, the BTG2 expression is high in quiescent thymocytes while expression decreases in proliferating progenitors, suggesting that the presence of BTG2 allows thymocytes to remain in a nondividing state (Konrad & Zuniga‐Pflucker, 2005). Similarly, BTG2 expression in mature T cells inhibits cell proliferation and survival (Ryu et al., 2014). BTG2 was also shown to negatively affect the expansion of HSCs in the bone marrow upon estradiol stimulation, by inhibiting the mammalian target of rapamycin (mTOR) pathway (B. C. Kim, Ryu, Oh, & Lim, 2008). Moreover, BTG2 favors RA‐induced hematopoietic differentiation through regulation of gene‐specific histone methylation, by recruiting the arginine methyltransferase PRMT1 to the RA receptor complex (Passeri et al., 2006).

An additional function for BTG1 and BTG2 in hematopoiesis was revealed with the identification of *BTG1* as a target of PAX5, a transcription factor that dictates the commitment of lymphoid progenitor cells to the B‐cell lineage (Schebesta et al., 2007). Moreover, BTG2 regulates pre‐B‐cell differentiation through PRMT1‐mediated methylation of CDK4, thus inducing cell‐cycle arrest to limit pre‐B‐cell expansion (Dolezal et al., 2017). In search of a functional role for BTG1 and its closely related family member BTG2 during B lymphopoiesis, we used knockout mice to study the fate of hematopoietic progenitor cells upon loss of these genes. Whereas the absence of *Btg1* and *Btg2* reduces the number of B‐progenitor cells in bone marrow and spleen, we demonstrated that both genes fulfill a unique role during the distinct stages of B‐cell development: loss of Btg2 affects the propagation of early progenitor cells (pre–pro, pro‐B and pre‐B cells), while Btg1 deficiency leads to deregulation of later stages of B‐lineage differentiation, including the immature B cells (Tijchon et al., 2016). In fact, Btg1 acts as a positive regulator of B‐cell progenitor outgrowth in response to IL‐7 using in vitro colony assays (Tijchon et al., 2016). Thus, depending on the cell lineage context, BTG1 and BTG2 can either enhance or inhibit cell proliferation.

### 4.4 Regulation of genotoxic stress response

BTG2 is required for DNA damage‐induced G2/M arrest, as the disruption of *BTG2* alters cellular response to DNA damaging agents (Rouault et al., 1992). In Hela cells, doxorubicin‐induced cell death is mediated by BTG2, which appears to involve the accumulation of H_2_O_2_ (Y. B. Lim, Park, & Lim, 2008). Conversely, BTG2 was shown to suppress apoptosis and promote DNA repair during DNA damage in response to p53 activation, suggesting that the apoptosis‐inducing effects of BTG proteins are context dependent (K. S. Choi et al., 2012).

Furthermore, BTG2 was identified as one of the genes upregulated in response to activation of p53, while the the loss of BTG2 expression cooperates with oncogenic Ras in the transformation of primary cells (Boiko et al., 2006). The tumor suppressor p53 and the proto‐oncogene Ras are among the most frequently mutated genes in human malignancies, and the cooperation between both regulatory networks to induce cellular transformation is well established. In primary fibroblasts, suppression of BTG2 mimics loss of p53 function in collaboration with oncogenic Ras (H‐Ras^v12^), allowing cells to bypass replicative senescence while triggering transformation and immortalization. Repression of BTG2 in this oncogenic setting raises the level of cyclins D1 and E1 and phosphorylation of Rb, which is in line with previous reports (Guardavaccaro et al., 2000; I. K. Lim et al., 1998). Further studies have identified additional crosstalk between the p53–BTG2 axis and oncogenic Ras, where BTG2, in the context of p53 deregulation, is capable of binding to H‐Ras^v12^ and repress its activity, while the perturbed function of BTG2 leads to elevated H‐Ras activity (Buganim et al., 2010). Recently, BTG2 was shown to regulate p53 activity via posttranslational modification. This BTG2‐mediated p53 regulation leads to a switch from senescence to apoptosis, which reduces tumorigenicity in bladder cancer cells expressing oncogenic Ras and mutant p53 (O. R. Choi, Ryu, & Lim, 2016). Altogether, these findings establish BTG2 as a tumor suppressor and show that its downregulation, as it is frequently observed in solid tumors, may synergize with oncogenic signals, such as Ras, to induce malignant transformation.

### 4.5 A role for BTG1 and BTG2 in the integrated stress responses (ISRs)

In the developing organism as well as in the adult, cells are exposed to a variety of stressors. These include extrinsic cell factors such as hypoxia or nutrient starvation, but also cell intrinsic stresses such as viral infections, oncogene activation or endoplasmic reticulum (ER) stress, which is the result of an accumulation of misfolded proteins in the ER. To optimally respond to these challenges and restore cellular homeostasis, eukaryotic cells have evolved an adaptive cellular mechanism known as the ISR. Activation of the ISR leads to a shutdown of global protein synthesis, which requires phosphorylation of eurkaryotic translation initiation factor 2 (EIF2α) by one of four stress‐activated kinases, which are selectively sensitive to either amino acid starvation, hypoxia, viral infection or protein misfolding. At the same time, however, translation of a select set of target genes, such as that of ATF4 is enhanced. Since ATF4 controls the expression of genes involved in amino acid transport and metabolism, protection from oxidative stress and protein homeostasis, increased expression or activation of ATF4 usually acts to promote cell survival and restore cellular homeostasis. However, under conditions of severe or sustained stress, activation of ATF4 may lead to the opposite effect, that is, the execution of apoptosis. As the expression of BTG1 and BTG2 are induced by a variety of stress stimuli that activate the ISR, we studied a potential role for BTG1 and BTG2 in ATF4‐mediated stress signaling. By exposing cells deficient for *Btg1* or *Btg2* to stressors that activate ATF4, it was observed that BTG1, but not BTG2, positively regulates ATF4‐mediated transcriptional activity. Moreover, we demonstrated that BTG1 physically interacts with ATF4, to modulate its activity by recruitment of the arginine methyltransferase PRMT1. Indeed, ATF4 is methylated by PRMT1 on several arginine residues, while the loss of ATF4 methylation appears to selectively reduce expression of ATF4 target genes implicated in apoptosis induction and stress‐induced growth arrest (Yuniati et al., 2016). As a consequence, cells deficient for BTG1 show increased cell survival under conditions of sustained cellular stress. Although these experiments point to a role for BTG1 as a transcriptional coregulator in the control of cellular stress responses, we cannot rule out that additional effects on stress signaling may involve posttranscriptional regulation by the Ccr4–Not complex. As for BTG2, the observation that its expression, in addition to its role in (p53 dependent) genotoxic stress, can also be induced by oxidative stress suggests different roles for this protein in cellular adaptation to stress. In response to stress challenges, such as serum deprivation and oxidative stress, the BTG2 expression is strongly upregulated as a consequence of the generation of reactive oxygen species (ROS) and subsequent activation of ΝF*‐κ*Β (Imran & Lim, 2013). ΝF‐*κ*Β signaling is not only central to inflammation and immunity, but also plays a role in oxidative stress through its crosstalk with ROS (Morgan & Liu, 2011). In this context, BTG2 may act as a downstream effector of ΝF‐*κ*Β in response to cellular stress.

## 5 BTG PROTEINS IN CANCER

### 5.1 Deregulated expression of BTG1 and BTG2 in solid tumors

Given the important roles of BTG1 and BTG2 in fundamental biological processes such as proliferation, differentiation, and cellular stress responses, it is not surprising that aberrations in expression or function of these proteins are observed in various malignancies. Deregulated expression of BTG1 or BTG2 is seen in a variety of solid tumors. In some of these cases decreased BTG1 expression appears to correlate with poor overall survival and tumor metastasis formation. The studies in gastric and hepatocellular carcinoma found no evidence for promoter hypermethylation or gene mutations as the causative factor for BTG1 downregulation (Kanda, Oya, et al., 2015; Kanda, Sugimoto, et al., 2015). However, BTG1 downregulation in ovarian carcinoma cell lines does appear to involve promoter methylation (J. Y. Kim, Do, Bae, & Kim, 2017). Overall, the early genetic event(s) that contribute to BTG1 loss in these solid malignancies are still largely unknown and remain to be determined.

In other solid tumor models, where *BTG1* is subject to posttranscriptional silencing by microRNAs, low BTG1 levels appear to be disadvantageous for cells. For instance, in colorectal carcinoma, *BTG1* was identified as a direct target of miR‐22, a class of miRNA controlling the switch between autophagy and apoptosis in response the chemotherapeutic agent 5‐FU (H. Zhang et al., 2015). The authors demonstrated that high levels of miR‐22 coincided with decreased *BTG1* expression, which rendered these colorectal cancer cells more sensitive to therapy. Likewise, downregulation of *BTG1* by miR‐454‐3p appears to increase sensitivity to irradiation in renal carcinoma cells by promoting cell death (Wu et al., 2014). On the other hand, overexpression of miR‐511 in human hepatoma cells and miR‐301A in colon cancer cells inhibits the expression of BTG1 and promotes tumor cell proliferation (He et al., 2017; S. Zhang et al., 2017). These studies not only reveal additional regulatory mechanism controlling BTG1 transcript levels but also suggest that the effects on cancer progression or response to therapy are highly cell and context dependent.

The role of BTG2 during tumor progression seems to be more unambigious. In breast carcinoma, BTG2 downregulation, through an unknown mechanism, leads to increased cyclin D1 expression and elevated AKT phosphorylation. Low level of BTG2 in breast tumor thus correlates with increased tumor grade, disease progression and decreased overall survival (Kawakubo et al., 2004, 2006; Takahashi et al., 2011; van de Vijver et al., 2002). An elevated amount of cyclin D1/cyclin E in liver cancer is a consequence of the low level of BTG2, leading to increased tumor grade (Z. Zhang et al., 2011). In prostate cancer, BTG2 is a target of miR‐32, miR‐21, and its suppression results in disease initiation and progression, therapy resistance, and metastasis (Coppola et al., 2013; Jalava et al., 2012). These findings point to a predominantly tumor suppressive function for BTG2. In vivo studies using knockout and overexpression of *Btg2* in mice confirm a role for this gene as a tumor suppressor in medulloblastoma (Farioli‐Vecchioli et al., 2007; Farioli‐Vecchioli, Cina, et al., 2012).

### 5.2 BTG1 and BTG2 are frequently affected by gene deletions and mutations in B‐cell malignancies

In the past decade, genome‐wide profiling studies revealed that genetic aberrations in *BTG1* and *BTG2* are frequently observed in B‐cell malignancies. Somatic missense mutations affecting either *BTG1* or *BTG2* are relatively common in diffuse large B‐cell lymphoma (DLBCL; Lohr et al., 2012; Morin et al., 2011; Reddy et al., 2017), while point mutations in *BTG1* and *BTG2* were also identified in follicular lymphoma (FL), a histologically low‐grade lymphoma (Pasqualucci et al., 2014). Of note, in the ABC subtype of DLBCL, genetic alterations in BTG1 are associated with poor survival, while in FL, mutations in BTG1 appear to be correlated with disease progression (Kridel et al., 2016; Reddy et al., 2017). In Burkitt lymphoma (BL) subtype with RBL2/p130 mutation, the BTG1 expression is suppressed, resulting in loss of growth control (De Falco et al., 2007). Finally, *BTG1* copy number alterations are detected with a high frequency in lymphoplasmacytic lymphoma cases, leading to speculation that *BTG1* loss may contribute to the pathogenesis of this non‐Hodgkin lymphoma subtype (Hunter et al., 2014). The mutations that are found in lymphomas most likely arise from erroneous somatic hypermutation (SHM) events. During B‐cell development, SHM, which is orchestrated by activation‐induced deaminase (AID), promotes antibody diversity and maturation. Off‐target AID activity, however, can also lead to mutations in adjacent oncogenes or tumor suppressors and contribute to lymphomagenesis. SHM‐associated mutation hotspots have been mapped closely to the regulatory region of *BTG2* and consequently may also be responsible for the introduction of mutations in this gene (Jiang, Soong, Wang, Melnick, & Elemento, 2012). Recent studies have shed new light on the role of BTG1 in lymphoma pathogenesis. By combining in vitro and in vivo studies, Li et al. (2014) demonstrated that MYC maintains a neoplastic state by suppression of four chromatin regulatory genes, one of which is BTG1. Suppression of these MYC effector genes involves upregulation of *miR‐17–92*. Knockdown of BTG1 alone was shown to be sufficient to (partly) overcome proliferation arrest in response to MYC inactivation, suggesting that suppression of BTG1, together with these other *miR‐17–92* targets, is required for MYC‐induced transformation and lymphomagenesis. Taken together, BTG1 appears to act as a negative regulator of proliferation and a tumor suppressor in lymphoma.

In B‐cell precursor acute lymphoblastic leukemia (BCP‐ALL), the most common type of cancer in children, microdeletions affecting *BTG1*, but not *BTG2*, are recurrently detected. Single copy losses affecting this gene occur at a frequency of around 9%, while BTG1 copy number losses appear to be enriched in specific cytogenetic subgroups, such as *ETV6‐RUNX1*, *BCR‐ABL1*, and *BCR‐ABL1*‐like positive BCP‐ALL, and co‐occur with genetic alterations affecting the B‐lineage determinants *IKZF1* and *PAX5* and the immune modulatory molecule *BTLA* (Kuiper et al., 2007; Mullighan et al., 2007; Roberts et al., 2014). We recently demonstrated that *BTG1* deletions arise as the result of off‐target V(D)J recombination, which takes place during pre‐B‐cell expansion to create a diverse repertoire of antigen receptor specific B and T cells (Waanders et al., 2012). Moreover, we observed that unique *BTG1* deletions can arise independently in multiple subclones, which either remain present as minor populations or develop into predominant clones. Interestingly, different leukemic blast populations lacking *BTG1* can be found as subclones during diagnosis and at relapse, confirming that BTG1 deletions occur repeatedly during disease progression (Mullighan et al., 2008; Waanders et al., 2012).

Although the origin and nature of BTG1 aberrations in BCP‐ALL have been elucidated, how BTG1 deregulation contributes to leukemogenesis is still not well understood. The presence of *BTG1* deletions at diagnosis suggests that loss of BTG1 most probably acts as a cooperating event during leukemic transformation (Moorman et al., 2012; Moorman et al., 2014). Indeed, we recently showed that in a mouse model, loss of *Btg1* cooperates with deletions of the tumor suppressor *Ikfz1* to promote leukemia development. Disease incidence increases while time‐to‐leukemia is shortened when either one or both copies of the *Btg1* gene are deleted (Scheijen et al., 2016). Furthermore, recent analysis looking at the co‐occurrence of *BTG1* deletion with specific genetic alterations has linked *BTG1* loss to the incidence of relapse. For instance, *BTG1* deletions predict a poor outcome in selected genetic subtypes of BCP‐ALL (Scheijen et al., 2016). In an independent cohort of relapsed pediatric BCP‐ALL, *BTG1* deletions were associated with induction failure/death and second relapse, specifically in high‐risk group patients. The combination of *BTG1* and deletions affecting *NR3C1*, which encodes the GR, appeared to be mutually exclusive and further increased the risk of death (Irving et al., 2016). Both of these studies are in line with our previous observations showing that loss of BTG1 confers resistance to synthetic glucocorticoids in cell culture models by modulating GR‐mediated gene expression (van Galen et al., 2010).

## 6 BTG1 AND BTG2 AS TUMOR SUPPRESSORS: CLINICAL IMPLICATIONS

A number of observations point to a tumor suppressive role for BTG1 and BTG2 in a range of malignancies, although in most examples, the exact mechanism by which BTG1 or BTG2 contribute to tumor development/progression requires further investigation. The fact that in solid tumors reduced expression of BTG1 or BTG2 associates with poor outcome suggests that the expression of their gene products could serve as biomarkers for disease progression. For example, BTG1 expression levels can be used to monitor the remission status of acute myeloid leukemia patients and the progression of proximal nondiffuse and diffuse gastric cancer patients (Cho et al., 2004; Kanda, Oya, et al., 2015). In addition, the finding that BTG1 deletions are enriched in distinct high‐risk ALL subgroups, such as *BCR‐ABL1*, and *BCR‐ABL1*‐like ALL, and are correlated with poor outcome in *IKZF1*‐deleted ALL (Scheijen et al., 2016), requires further investigation to carefully examine the specific contribution of *BTG1* copy number losses to disease progression in these ALL subtypes.

In breast and prostate carcinomas, where BTG2 expression was found to be reduced, therapies restoring BTG2 expression may contribute to the inhibition of cancer cell proliferation. Examples of such interventions include the ErbB/HER inhibitor lapatinib in breast tumors, the therapeutic radionuclide iodine‐131 in thyroid cancer cells, and the chemotherapy drug cisplatin, the cell‐cycle inhibitor l‐mimosine, and the topoisomerase inhibitors camptothecin and doxorubicin in prostate carcinoma cells (Chiang, Tsui, Chung, Yeh, Chang, et al., 2014; Chiang, Tsui, Chung, Yeh, Feng, et al., 2014; Chung et al., 2012; Takahashi et al., 2011; Zhao & Pang, 2017). Moreover, BTG2 is also among a set of genes whose expression pattern can be used as a biomarker to predict the recurrence of prostate cancer (Long et al., 2014).

## 7 CONCLUSIONS AND PERSPECTIVES

BTG1 and BTG2 are highly versatile proteins that exhibit both unique and redundant roles in growth control, differentiation and the regulation of apoptosis. Moreover, both proteins are involved in metabolic regulation and adaptation to cellular stress. While deregulation of BTG1 and BTG2 is observed in a variety of malignancies and often associated with an unfavorable prognosis, their complex roles during malignant transformation and disease progression require further investigation. Also with respect to their molecular functions, a number of questions remain unanswered. While some studies emphasize the role of these proteins as regulators of mRNA deadenylation, affecting mRNA stability or even protein translation, BTG proteins also function as transcriptional coregulators, affecting arginine methylation of transcription factors and histone proteins. In this respect, the association of PRTM1 with BTG1 or BTG2 will promote the methylation of specific subsets of target proteins. PRMT1 has many substrates, which explains why loss‐of‐function studies have shown profound, yet tissue‐specific and heterogenous effects (Bedford & Clarke, 2009). It frequently remains unclear which substrate is responsible for a specific phenotype that can be found in studies of PRMT1 deficiency. As discussed above, loss of BTG1 or BTG2 can be associated with the promotion of tumor growth. At least part of this phenotype may be attributable to the loss of PRMT1‐mediated methylation of specific substrates. This is why it is important to study PRMT1 function in complex with cofactors and their substrates.

The fact that BTG proteins are upregulated in response to a broad variety of cellular stressors, growth factors, and steroid hormones, suggest a central role in maintaining cellular homeostasis. However, an overarching mechanism has yet to be identified. A comprehensive understanding of how downregulation/inactivation of these versatile proteins affects tumor progression or response to therapy may ultimately contribute to the design of novel and more effective anticancer therapies.
